# Conversion practices in Aotearoa New Zealand: Developing a holistic response to spiritual abuse

**DOI:** 10.1371/journal.pone.0302163

**Published:** 2024-05-01

**Authors:** Michael Roguski, Nicola Atwool

**Affiliations:** 1 Kaitiaki Research and Evaluation, Wellington, New Zealand; 2 Victoria University of Wellington, Wellington, New Zealand; 3 Nicola Atwool Consulting, Dunedin, New Zealand; Ateneo de Manila University, PHILIPPINES

## Abstract

Research arising from conversion practices, also known as conversion therapy and sexual orientation and gender identity change efforts, has generally been underpinned by an emancipatory discourse that has evolved to counter harmful practices by evidencing associated harms and estimating prevalence. Little attention, however, has focused on what is required to support survivors, inclusive of those currently or those having previously experienced conversion efforts. Within a context of Aotearoa New Zealand having recently criminalised conversion practices, this study adopted an in-depth qualitative research design, informed by a dual adherence to life history and an empowerment methodology. Twenty-three religious conversion practice survivors, who had experienced religious conversion practices across a range of Christian identified faith settings, were interviewed. Participants had a median age of 34 and the majority identified as New Zealand European, cisgender, and gay. Participant narratives were discursively analysed. Three primary discourses were identified that inform the needed development of interventions and supports: 1) pervasive framing of conversion practices as harm, rather than spiritual abuse, has minimised the impacts of conversion practices. Rather, conceptualising conversion as spiritual abuse positions conversion practices as requiring urgent intervention and ongoing support, inclusive of the development of policy and operational responses; 2) the coercive nature of spiritual abuse needs to be appreciated in terms of spiritual, social, and structural entrapment; 3) the metaphor of a pipeline was enlisted to encapsulate the need for a multidimensional array of interventions to ensure those entrapped within spiritual abuse have a “pipeline to safety”. Holistic survivor-centric conversion-related responses to spiritual abuse are required. These need to be informed by an understanding of entrapment and the associated need for holistic responses, inclusive of extraction pathways and support for those entrenched within abusive religious settings, support immediately after leaving abusive environments, and support throughout the survivors’ healing journeys.

## 1. Introduction

Conversion therapy has been popularised as denoting efforts to alter a person’s sexual orientation to align with heteronormative ideals; ideals purported by faith-informed dictates, beliefs, and practices. More recently the field has evolved in focus, inclusivity, and complexity to encompass gender identity and gender expressions that counter heteronormative and traditional cisgender conceptualisations [1, 2]. Underpinning the field’s complexity is a lack of definitional consensus and conversion-related research has been inconsistent in its inclusivity; differing according to the inclusion of sexual orientation and gender diversity; a dilemma founded in the field’s fledging development. Moreover, while conversion-related research has generally focused on conversion within a religious context, the field has expanded to sometimes include non-religious conversion, inclusive of attempting to change or suppress sexual orientation or gender identity in healthcare settings, such as withholding access to gender-affirming healthcare [1, 3].

Despite these challenges, conversion-related studies have discursively shared an emancipatory focus, centred on conversion-related harms and prevalence, to evidence the need to eradicate conversion practices. Although piecemeal, the research has documented a host of harms that impact on a significant number of people whose sexuality, gender identity or gender expression exist external to heteronormative and conventional faith-based dictates. Within this context, evidence has spearheaded a small but growing number of nations who have restricted the administration of conversion therapies. For instance, as of 2023, only eleven United Nations Member States have enacted nationwide conversion-related laws. These jurisdictions include Aotearoa New Zealand, Belgium, Canada, Cyprus, Ecuador, France, Germany, Greece, Iceland, Malta, and Spain. It is therefore understandable that researchers and community advocates continue to focus on the harm and prevalence of such practices.

Having recently criminalised conversion therapy with the passing of the Conversion Practices Prohibition Legislation Act 2022 [4], the experience of survivors in Aotearoa New Zealand provides an opportunity to move from an apologetic frame to addressing the types, and kinds, of supports survivors require in a post-criminalisation environment.

### 1.1. Framing conversion practices

The field’s evolution was founded within western psychological epistemologies that pathologised same sex attraction and resulted in the adoption of a deficit-reparative focus which explored the efficacy of “conversion”, “reparative” or “reorientation” therapies [5]. Reparative “therapeutic” methods ranged from psychotherapeutic interventions, including hypnosis and psychoanalytic techniques [6, 7]; behavioural therapeutic methods including masturbatory reconditioning and aversion therapy [8–10] abstinence training and teaching traditional gender roles [6, 7], and biological methods, such as medication, electroconvulsive therapy, surgery (castration, lobotomy, and the removal of ovaries), and hormone therapy [7, 11, 12].

In 1973, growing concern over the harms associated with conversion therapy, and especially driven by gay liberation activists [13], culminated in the American Psychological Association’s removal of homosexuality from the Diagnostic and Statistical Manual of Mental Disorders; a move that signalled a significant epistemological shift unseating the psychological legitimacy of reparative therapy [14].

Western psychology’s gradual movement away from a reparative focus coincided with the increasing influence of the religious conversion therapy movement [15]. These therapies have persisted, justified by theological arguments that position sexual orientation, gender identity, and gender expression that fall outside of religious dictates of heteronormativity as moral transgressions and supported by proponents’ claims that the combination of faith and the use reparative psychotherapeutic interventions can realign the individual to fit heteronormative ideals [1, 2].

Within this context, a singular focus on conversion therapy failed to encompass survivors’ varied experiences and limited the extent to which survivors’ experiences can be understood. In response, some studies broadened conversion-related conceptualisations to include *conversion practices* that acknowledge the existence and impact of *formal conversion practices*, generally regarded as synonymous with conversion therapy and inclusive of reparative “therapeutic” methods, and *informal conversion practices* which acknowledge the existence and associated harms of informal change efforts, inclusive of negative change messaging from friends, family, faith leaders, and community members [2]. Significantly, more recently, a small number of conversion practice-centred studies have included *conversion ideology* to umbrella conversion practices within heteronormative and cisgender discourses, manifesting as overt spoken beliefs and teachings, and the underlying culture of a particular community of people, that stress the ‘brokenness’ of those with non-conforming gender and sexual identities while simultaneously asserting that spiritual or psychological interventions can “heal” an individual [2, 16].

Parallel to the use of *conversion practices*, a growing number of researchers have referred to ‘sexual orientation and gender identity or expression change efforts’ (SOGIECE). However, the use of SOGIECE lacks clear definitional parameters and, consequently, research findings are not comparable. Some studies have treated sexual orientation change efforts (SOCE) as synonymous to conversion or reparative therapy [1, 5, 17, 18]. Others have treated SOGIECE synonymously with informal conversion practices and the impact of institutional cisgender heteronormative dictates [19] whereas others invoke SOGIECE to include conversion practices and conversion ideology [20]. Finally, some SOGIECE-related studies have included change efforts external to faith-based contexts, a development that is important and reinforces the need for conceptual consensus [1].

To reduce definitional ambiguity the current study is framed in accordance with faith-based conversion practice conceptualisations, inclusive of conversion ideology and formal and informal conversion practices.

### 1.2. Conversion practices research

The advent of the religious conversion therapy movement coincided with three primary tranches of emancipatory-centred studies that aimed to provide an evidence base to support the eradication of conversion practices. The first research tranche focused on harms arising from efforts to change someone’s sexual orientation, gender identity or expression. Identified harms have included fear and shame [21], anxiety [22], depression [21–23], negative self-esteem [21], suicidality [1, 3, 17, 21, 23, 24], non-suicidal self-injury [1, 3, 22], and self-hatred [21–23, 25], as well as internalised homonegativity [21, 23], sexual dysfunction [21], decreased capacity for same-sex intimacy [23], significant harm to interpersonal relationships and social functioning [21, 23] and spiritual harm, inclusive of a loss of faith and spirituality [21, 23, 25].

A second tranche of research has focused on conversion practice prevalence [1, 3, 17, 19, 24, 26–31]. The majority of prevalence surveys to date have been conducted in the United States and have included adult populations. Using a phone survey, Blosnich et al. [17] estimated that 7% of transgender adults had experienced any conversion therapy, while James and colleagues [26], using an online survey, estimated that 14% of transgender adults had experienced conversion therapy from family members. An online survey of LGBTIQ+ youth conducted by Green et al. [24] reported that 4.2% had experienced conversion therapy. Meanley et al. [28] reported that 17.7% of middle-aged and older men who have sex with men, who were included in the US multicentre AIDS cohort study, had experienced conversion therapy.

There are wide variations in prevalence estimates from investigations conducted in Aotearoa New Zealand. An online survey of gender and sexual diverse youth estimated that 3.7% had experienced conversion therapy [1]. In contrast, Veale et al. [3] estimated that 19.7% of trans and non-binary adults aged 14 years and over had experienced conversion therapy. Differences in prevalence estimates are likely to be due to the age of the population sampled, with older populations having increased opportunity for experience, the gender or sexual orientation of participants, recruitment methods, and community context. For example, a face-to-face survey conducted by Ryan and colleagues in the United States, recruiting youth participants from bars and clubs [30] found that over 50% of their participants had experienced conversion therapy from family members, and around half of these had also experienced more formal conversion therapy. Notably, reliable prevalence data has been difficult to access for lesbian or bisexual cisgender women as gender has not been disaggregated from cisgender male experiences [24].

A third, and most recent, tranche of research has explored what has helped survivors in their recovery. In general, commentary has centred on therapeutic support interventions and have either documented mental health professionals’ experiences in working alongside survivors and how these experiences can inform clinical practice [14, 32], or have involved phenomenological investigations into survivors’ experiences with therapy, with a particular focus on how mental health professionals should align practice to the lived experiences and needs of survivors [22, 23, 33].

In light of the scant evidence available to mental health professionals, Jones et al. [20] carried out a qualitative study of survivors in Australia to better understand survivor recovery pathways. The authors found that post-conversion practice recovery was more successful if survivors experience three provisions: people who are affirming, enabling them to freely be themselves–especially health and mental health practitioners, family and friends, and survivor support groups; considerable time and internal motivation to enable support to be effective; and conflicting aspects of identities and beliefs are reconciled in ways that foreground survivors’ autonomy in their reconstruction. Despite the use of an ecological model to frame the research, the study’s strong mental health focus, in general, restricted recovery experiences within the purview of mental health professionals and not wider support mechanisms, either in the short or longer-term.

### 1.3. The current study

Conversion practices research has generally adopted an emancipatory focus by documenting harms, prevalence or informing appropriate therapeutic approaches to support survivors. Little attention has focused on what is required to support survivors, external to mental health responses, inclusive of current, past, and potential exposure to conversion practices. The current study seeks to understand holistic survivor-centric intervention opportunities and support needs. Notably, no support-focused research has been undertaken in nations after the criminalisation of conversion practices, and the Aotearoa New Zealand context provides an opportunity to understand survivor needs in a context of legislated protection.

## 2. Materials and methods

An in-depth qualitative research design was employed informed by a dual adherence to life history and an empowerment methodology. Life history methodology was used to guide the collection and interpretation of narratives to understand individuals’ intricate life journey experiences within social, faith, cultural, and historical context [34]. Empowerment methodology explicitly positions participant voices as central, valid, and reliable whilst simultaneously acknowledging the socio-political history of those whose sexual orientation, gender identity, and gender expression exist in opposition to heteronormative dictates [35, 36].

### 2.1. Recruitment

Ethics approval for this study was granted by Aotearoa Research Ethics Committee (approval number: AREC23_04). Participants were recruited using purposive sampling between 10 March and 28 April 2023. The New Zealand Human Rights Commission commissioned the study and promoted the research through social media posts, radio, and sharing study-related information through its survivor networks. In addition, research team members shared an invitation to participate through their networks. Participants were eligible to take part if aged 18 years and over and if they identified as a conversion practice survivor, inclusive of clinical and faith-based settings. If interested, potential participants were invited to contact MR who provided an information sheet, consent form, and biographies of four interviewers that participants were invited to select to carry out the interview. Participants were also informed about the availability of post-interview psychological support. Best efforts were made to provide a range of interviewers according to gender (cisgender, transgender, and non-binary), sexuality (gay, lesbian, gender queer, and takatāpui), age (between 40 and 72 years), ethnicity (Māori and New Zealand European), and whether the interviewer identified as a survivor. Written consent to participate in the study was obtained from all participants (see Table 1 for a glossary Māori translations).

**Table 1:** Glossary.

Te Reo Māori	English Translation
Hapori	Community
Hapū	constellations of whānau
Hauora hinengaro	mental health
Hauora tinana.	physical health
Hauora wairua	spiritual health
Iwi	Tribe
Koha	Gift
Māori	Indigenous people of Aotearoa
Takatāpui	commonly defined as intimate companion of the same sex and encompasses Māori of diverse sexualities, genders, and sex characteristics
Whānau	extended family

### 2.2. Participants

Twenty-five people participated in the interviews. Of these 23 people reported experiencing religious conversion practices across a range of Christian denominations, such as Anglican, Brethren, Roman Catholic, and Pentecostal, as well as independent communities of faith. The remaining two participants were not included in this study and their narrative will be included in a separate paper on non-religious conversion practice experiences.

Participants’ ages ranged between 24 and 72 years, with a median age of 34. Most participants identified as New Zealand European / European (n = 15) with the remaining participants identifying as Asian or Southeast Asian (n = 4), Māori (n = 2) or Pasifika (n = 2).

Cis males were overrepresented in the sample (n = 13). The remaining participants identified as transgender (n = 3), cis female (n = 3), non-binary (n = 3), and gender queer (n = 1). In addition, one participant reported being born with a variation of sex characteristics but did not identify as intersex, describing themselves as transgender. The majority of participants identified as gay (n = 8) with remaining participants identifying their sexuality as bisexual (n = 4), lesbian (n = 3), pansexual (n = 2), gender queer (n = 2), cisgender takatāpui (n = 2). Two participants stated that they did not have a sexuality.

Approximately three quarters (n = 18) of the participants had experienced formal conversion practices. Practices included aversion therapy, counselling through licensed counsellors endorsed by the faith setting or via members of the faith setting who were not formally trained in counselling, medicalisation by physicians endorsed by the faith setting, chemical castration, and through attendance at a variety of multiple-week group conversion sessions derived from US ‘therapeutic’ interventions, such as exorcism or deliverance within a therapeutic setting, and counselling, characterised by a formalised structure and accompanied by individually guided workbooks. Occurring parallel to these processes were a range of mechanisms that repeatedly othered the individual within the faith community, inclusive of public confessions of sin, being sent overseas, and threats of excommunication and punishment of the individual’s family.

The majority of participants first engaged in formal conversion practices in their mid- to late teens; generally occurring before leaving home. Significantly, one participant first engaged in formal conversion therapy at seven years of age, one at 12, and two at 14 years. A minority of participants first engaged in formal conversion practices after leaving home and had either attended Bible college or had accepted leadership roles in a faith setting outside of their town or city of origin. For these participants, encouragement to engage in formal conversion practices occurred because their social network was predominantly or exclusively people who adopted literal biblical heteronormative perspectives regarding sexuality and gender. One participant engaged in conversion practices after they attended university, despite members of their social network urging them to desist.

Some participants engaged in formal conversion practices for a few weeks, others described being in therapy for several years. Some survivors described consecutive involvement in formal conversion practices whereas others described episodic engagement over a period of months and then shifting to informal practices only to return to more formal practices later. In these situations, some years would pass before the individual would reengage in formal conversion practices.

All participants experienced informal conversion practices (n = 23). These practices included prayer, pressure from peers, group deliverance within a congregational context, and non-therapeutic pastoral mentoring that was indirectly associated with negative change messaging, such as engaging in “gender appropriate” activities. Notably, all participants had experienced variations of conversion ideology.

### 2.3. Data collection

Due to the ongoing impact of COVID-19, individual interviews were conducted online by one of four research team members in the second quarter of 2023.

**Fig 1:**
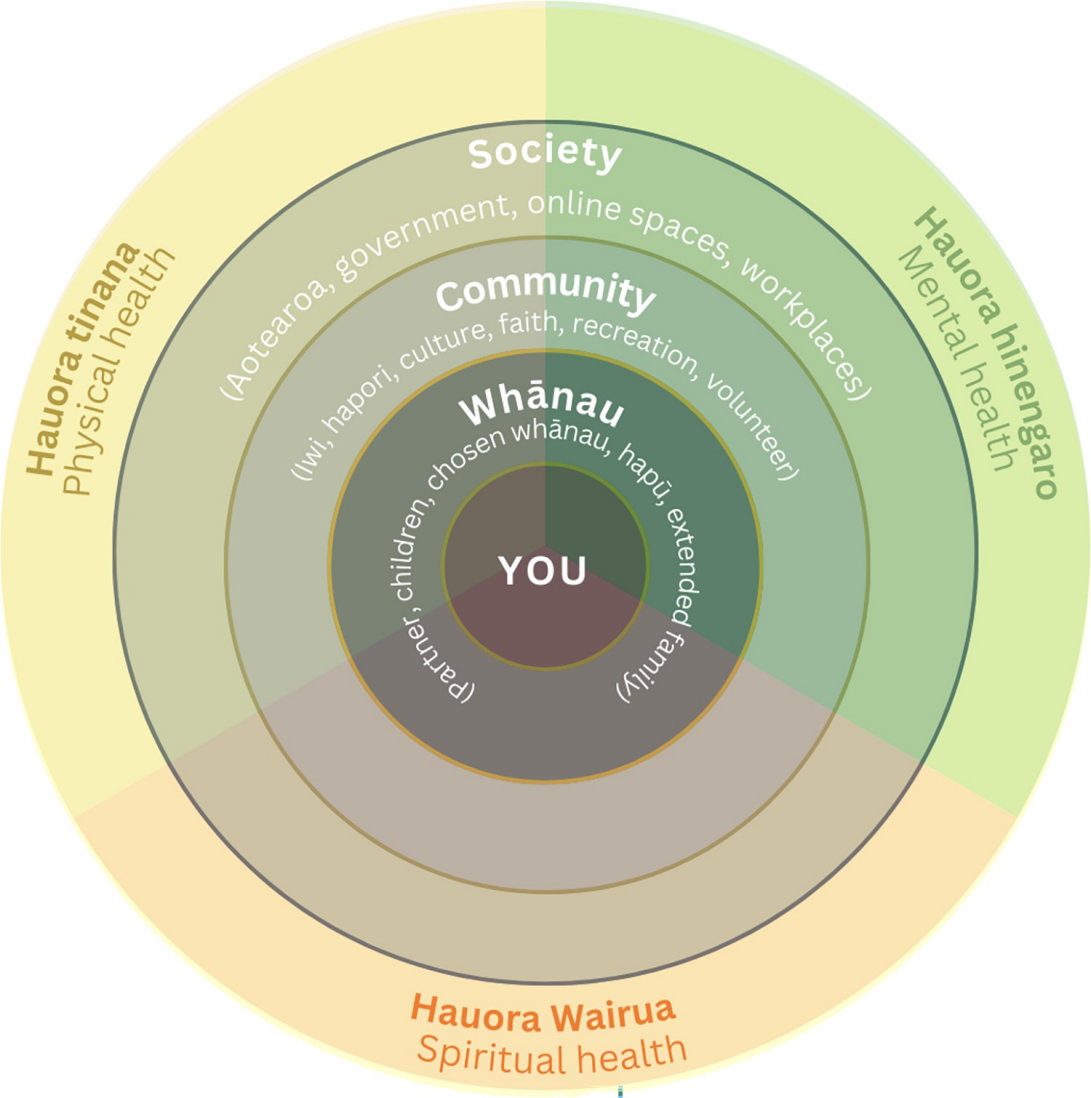
Visual interview prompt.

Interviews followed a semi-structured format that was accompanied by a visual prompt that had been designed in collaboration with a survivor-led advisory group to elicit holistic responses (see Fig 1). The visual prompt positioned the survivor in the centre of five concentric circles. Emanating outwards were whānau (partner, children, chosen family, friends, hapū, extended family), community (Iwi, hapori, culture, faith, recreation, volunteering), society (Aotearoa, government, online spaces, workplaces). The final circle positioned, hauora tinana (physical health), hauora wairua (spiritual health), and hauora hinengaro (mental health) as equidistant.

Interviews began by participants being asked a series of demographic questions (age, gender, sexual orientation, and whether the individual had been born with a variation of sex characteristics). Participants were then asked about their conversion practice experiences, impacts, what has helped, obstacles to the individual’s recovery, the kind of support that would have been useful when the individual first decided to reject conversion practices, the type of support the individual currently needs, and what needs to be done in terms of policy, legislation, and societal attitudes.

The interviews ranged between 60 and 90 minutes and were audio recorded and transcribed with participant consent. Participants received a $50 koha (gift) at the interview’s conclusion, in recognition of their time commitment.

### 2.4. Data analysis

A discursive approach underpinned transcript analysis. As described by Fairclough [37], this process involved developing emergent themes focused on the presence and interplay of ubiquitous discourses. The process involved attending to the participants’ context, focusing on the language participants used to describe their experiences, and centring on the emergent discursive concepts. Emerging discourses were consistently examined to determine the extent to which they were common across participants. In practice, this meant that discursive positions were created within an analysis framework. The analysis was initially conducted by MR and then refined further by NA. Complementing life history and empowerment methodology the analysis draws heavily on deidentified verbatim quotes that illustrate the emergent discourses.

## 3. Findings

### 3.1. Discursive context—contextualising experiences of trauma and healing

A common impediment to participants’ healing journeys was a minimisation of the insidiousness of conversion practices and the pervasive impacts of conversion practice trauma. Minimisation occurred across multiple societal levels, inclusive of faith-based environments, helping professionals, and post-conversion practice social networks, and was compounded by non-survivors’ lack of knowledge about the complex dynamics underpinning conversion practices, and assumptions that such dynamics are a natural consequence of membership in a faith community. Above all, participants stressed that minimisation occurs because of a failure to appropriately frame conversion practices as spiritual abuse.

Multiple participants reinforced the need to elevate conversion practices to the level of spiritual abuse and invoked similarities between the dynamics of family violence and conversion practices. Whilst family violence literature frames perpetration of violence within coercive power dynamics [38], participants described spiritual abuse as a form of coercive control enacted by those in a position of power within, and associated with, a faith-based setting, whether formal, such as conventional faith settings, or informal faith-based collectives. The mechanics of spiritual abuse include the use of faith-based teachings, threats, and demonisation to coerce and cause harm to the individual. Significantly, spiritual abuse can include the use of emotional, financial, physical, psychological, medical, and sexual abuse and these were common across the contexts in which participants experienced conversion practices.

Similarities also emerged between participants’ varied accounts of being “trapped” and the need to locate coercive control within a broader understanding of entrapment [38, 39]. Tolmie et al. [39] describe intimate partner violence operating as a form of social entrapment which comprises three dimensions: social isolation, fear and coercion that the perpetrator’s coercive and controlling behaviour creates in the survivor’s life; the indifference of powerful institutions to the survivor’s suffering; and the exacerbation of coercive control by the structural inequities associated with gender, class, race and disability. The intersection of the latter manifestations of social entrapment are commonly understood as structural entrapment. Within this context, participants’ experiences of being trapped are acknowledged as manifestations of spiritual entrapment. Much like intimate partner violence, the spiritual abuse described by participants included social isolation, fear and coercion, an indifference of a powerful institution (the faith community), and the compounding of structural inequities experienced by sexual and gender diverse people.

Participants equally stressed that spiritual abuse needs to be appreciated as occurring in environments of harm, reflective of structures that place excessive controls on their members and requiring unwavering devotion to a set of beliefs and inerrancy which were used to other those who fall outside of biblical dictates. Collusion was described as integral to these environments whereby members of the faith community, including family members, collude to change the individual’s sexual orientation, gender identity, and gender expression. Within this vein, environments of harm were commonly referred to as “cults” or “cult-like”, a metaphor that was used across participants regardless of the context in which the conversion practices occurred.

*They [fundamentalist faith settings] are cult-like if not actual cults. There are so many barriers to getting out of it; getting into a position where you feel free to leave. (Participant #1)*

Complementing the pervasive impact of faith-based environments of harm, participants stressed that it is erroneous to frame conversion practices external to conversion ideology. Effectively, conversion practices exist within environments of harm that negatively frame spiritualised categorisations of those who fall outside of prescribed sexual and gender norms as sexual perversions, inclusive of homosexuality, paedophilia, adultery, and masturbation; all of which the individual can be cured of through the Word of God. Within this context, participants stressed their entry into formal or informal conversion practices had been facilitated by a consistent exposure to deviantised othering.

*Conversion practices are pervasive and that is where most damage gets done. I mean, there’s the explicit conversion stuff, but there’s all of what leads you to be vulnerable to that, which is the message that you’re not acceptable the way you are. And that includes the church environment, Christian schools, and the home as well. (Participant #2)*

Insidiously, and complementing the metaphor of a cult, participants described that failure to comply with communities of faith directives resulted in their punishment and expulsion. Significantly, narratives positioned the survivor as a contagion, someone to be shunned by members of the faith community, including friends and family or, at the least, having stringent limits placed on interactions. The discourse of contagion is significant as it encapsulates dis-ease and the pervasive, and persistent, impacts.

*My parents and brother don’t know where I live. We can’t give them my address because they send hate mail. Well, they send loving messages of how we’re going to go to hell. My partner and I, my son came out as trans five years ago and they cut him off completely. The other thing, because my son came out, I went down a level myself. They could still tolerate me being around their children, my nieces and nephews but once my son came out, I’d become someone who perverted children. And the Bible says that if anyone hurts one of my children, they should have a stone put around their necks and be flung into the sea. As far as they’re concerned, that’s what I am. So now I have zero contact with 12 or 13 of my nieces and nephews. (Participant #3)*

The following analysis is structured according to four primary discourses. The first three discourses frame survivor experiences in terms of primary barriers to either identification of experiences as abuse or impediments to the survivor’s healing journey. These include (1) spiritual entrapment, (2) social entrapment, and (3) structural entrapment. A fourth discourse, pipelines to safety and recovery, presents survivor-derived suggestions for survivor-centred intervention and support.

### 3.2. Spiritual entrapment

Within the cultural dynamics of the faith setting, survivors had been unable to identify conversion practices as abuse; a dynamic that is understandable considering fundamentalist teachings and having been socialised to obey faith leaders’ dictates as emulating “God’s voice on Earth”. Rather than consent, participants stressed they were entrapped in environments that demanded obedience; survivors surrendering choice in lieu of the faith setting’s authority over the individual.

*And I think it’s important to say that I did go along with all of this. And I wasn’t forced to take that prescription [chemical castration]. I wasn’t forced to take the drugs. But this was in the context of the church, the leader is God’s voice on Earth. And everything he says is God literally speaking through a man. And he can do no wrong. And so, if you disobeyed the leader, then that was equivalent in many ways to disobeying God. To a young teenager, who’s grown up in this belief system, it was utterly unthinkable that you would disobey what the leader was saying. And if the leader tells you to see a doctor, and then the doctor tells you to take these pills, it’s not even an option to say no because you’d be disobeying God. (Participant #4)*

Common to participants’ experiences of spiritual entrapment were examples of psychological abuse where the survivor’s ability to make an informed decision was questioned, either because they were told that they were mentally unwell or they were being influenced by nefarious forces, such as demonic possession. Such manifestations of entrapment were enacted when survivors communicated that they were contemplating either disengaging from formal conversion practices or leaving the faith setting.

*When I told the priest that I was leaving the church, they refused to accept it, and they told me that I was mentally unwell. (Participant #5)*

Associated with emotional and psychological abuse, participants described examples of punishment, either the possibility or enactment of a threat, which eroded individual agency and reinforced the individual as subservient to those in power. Discursively, these threats commonly reflected a discourse of contagion, whereby the individual had to be removed from the faith community, either physically or symbolically, such as loss of leadership positions, so to contain transmission. Notably, punishment often coincided with the individual’s family being punished; a situation that resulted in considerable guilt for the survivor.

*And the crunch point for them [church elders] was when I came out to my siblings, which for them [church elders] was a step too far. After that I was put in the first stage of excommunication, which meant that all members of the church were banned from speaking to me and I was banned from attending church services. That first stage feels like a form of purgatory where you are extremely isolated. (Participant #4)*

An especially insidious form of entrapment involved some survivors being removed from their faith communities and sent either overseas or to another location within the country for “intervention” or “treatment”. In all cases, the individual’s placement in unfamiliar settings resulted in heightened levels of isolation and a complete reliance on those with whom they had been “placed” that prevented them from leaving an abusive environment.

*Where was I going to go? I never tried to escape, but I didn’t have a phone. I was staying with church people. They were there every waking moment of the day. I didn’t have a credit card and I had no access to communications, no access to the outside world. Assuming that I could have left where was I going to go? (Participant #6)*

### 3.3. Social entrapment

Spiritual entrapment was framed as pervasive, extending beyond the rejection of conversion practices and extrication from abusive faith settings. Formal and informal conversion practice survivors described having lived cocooned existences that had prevented them from engaging in developmental tasks commonly navigated by their secular peers; thereby failing to acquire the life and interpersonal skills needed to function independently as adults, external to the faith environment.

*I was six or seven years behind everyone else developmentally. My whole life had been the community. That was all taken away from me—all those people I’d relied on for my guidance, my support. I lost everybody and everything as a 19-year-old. (Participant #7)*

Discursively, these experiences reflected variations of social entrapment, denoted by the individual’s extreme isolation and lack of knowledge about how to navigate life external to the abusive faith setting. Commonly cited barriers to engaging in recovery were homelessness, a lack of employable skills, and a lack of financial literacy.

*The aftermath of when you are kicked out is horrendous. I didn’t really know what was normal and what wasn’t. There are so many practical things that you have to deal with that you don’t fully have the scope to deal with. I’d never had to cook for myself. I’d never had to do all number of things. And I’d never watched TV. I’d never eaten in a restaurant. I didn’t know how to go to the pub. Suddenly I was in a situation where all of these things that to most people are very mundane, everyday occurrences were brand new to me and they took a huge amount of psychological labour and the labour of learning those new processes was so overwhelming. (Participant #8)*

Significantly, participants’ social entrapment was aggravated by heightened levels of vulnerability, anxiety, suicidality, self-loathing, and identity confusion that accompanied their exit from faith communities: the internalisation of cis-heteronormative messaging acting to prevent either entry to, or authentic integration within, “queer” networks.

*I had months of feeling like an absolute failure because I hadn’t been able to change. I was now going to live this life that wasn’t fully satisfying, a life of misery or whatnot. I just thought, well, that’s better than what I was experiencing before. (Participant #9)*

Finally, some participants shared having been financially entrapped, having acquired student loans to pay for their Bible college studies and being required to pay for these following their expulsion. These instances of financial entrapment were especially difficult for the survivors as the loans had implicitly funded their formal conversion practices and the required repayments had a detrimental impact on their ability to live independently.

### 3.4. Structural entrapment

Various manifestations of structural entrapment, reinforced by institutional inaction and non-inclusion through legislation or the lack of provision for intervening and supporting spiritual abuse survivors, were identified as creating significant barriers to the individual leaving abusive religious settings or the individual’s healing, post-conversion practices.

#### 3.4.1. Inadequacy of legislation

Recently enacted anti-conversion practice legislation was unanimously viewed as inadequate. While participants acknowledged the symbolic importance of the legislation, in that it clearly demarcates the unacceptability of formal conversion practices, they were sceptical that the law, as a singular intervention, is sufficient to end conversion practices.

On one level participants were sceptical because some fundamentalist faith settings were reported to have developed covert ‘therapeutic’ strategies to circumnavigate legal categorisations or operating such practices “underground”.

*I’ve got no doubt in my mind that it still goes on. The law changing the rules on conversion therapy is not going to stop it because it’s going to be pushed underground which is going to get more dangerous because it’s not going to be talked about. (Participant #11)*

The legislation was also critiqued for placing the onus on the victim to report breaches of the law, a requirement that was regarded as problematic on two levels. First, reporting requirements were regarded as unrealistic as the majority of formal conversion practice survivors had “willingly” participated in the various “therapeutic” interventions, and survivors only recognised their abuse as conversion practices after they had left the abusive environments, often after a number of years. Next, reporting spiritual abuse is problematic as it commonly occurs in settings where friends and family, who are members of a community of faith, have colluded with the various mechanics of abuse. Within this context a desire to avoid the possible criminalisation of the survivor’s loved ones was regarded as a significant barrier to reporting, a dilemma that has been identified in other abuse settings [36, 40–43].

Finally, the legislation was viewed as inadequate because it does not address informal and conversion practice ideology. As a consequence, outside of formal therapeutic environments, those who fall outside of heteronormative definitions of “appropriate” continue to experience harm, inclusive of gender identity, gender expression, and sexual identities. Especially noted mechanisms of conversion ideology included continued othering through theological and spiritualised dictates, deliverance, calls for members of the faith to seek prayer for breaches of heteronormative norms, and excommunication.

*The law is a pretty weak kind of instrument. When you are up against a cult with that level of fundamental belief in the rightness of what they’re doing, there is a need for laws that protect people beyond just conversion therapy. I mean it’s nice that they’ve changed the law, but it does nothing. (Participant #7)*

### 3.5. Lack of systemic intervention and support

A lack of systemic support was voiced as contributing to the individual’s entrapment within abusive religious structures and placing the individual at increased levels of vulnerability; this was especially noted in reference to faith-run schools and those who are homeschooled, whereby students are shielded from exposure to alternative discourses that legitimise otherness. Moreover, in the absence of interventions that can support an individual to leave an abusive environment, and reinforced by legislation that places the individual under the protection of the family, many victims are forced to remain in environments of harm.

*There are always going to be lonely kids lost in churches who have no way out. Their only option is to wait until they are old enough to leave. If you are 12 and being told that you are evil, that means you have six years ahead of you until you are old enough to leave to go to university. It’s either wait and live in hell or live on the streets. That’s a terrible place to put a young person in. When I was that age, I thought suicide was my only option. (Participant #11)*

#### 3.5.1. Medical and psychotherapeutic support

Medical and psychological support was essential to participants’ recovery. Medical providers were especially noted as pivotal as they were often the first professionals contacted and are an essential component of a referral pathway.

The majority of participants described having lived with a host of mental health challenges, such as anxiety, addictions, depression, dissociative personality disorder, obsessive compulsive disorder, and post-traumatic stress disorder (PTSD), arising from their formal and informal conversion practice experiences. Prolonged periods of suicidality were the most commonly referenced. These periods varied in length, and only abated when the individual was able to consolidate a positive sense of identity, a process referred to by Beckstead and Morrow [23] as congruence. For some, however, mental health challenges did not abate and presented as a lifelong challenge.

In general, participants described finding appropriately informed medical and mental health support extremely difficult. Participants commonly described disappointment when accessing support because clinicians lacked an understanding of the effects of living in cult-like structures, had no or limited understanding of spiritual abuse, and lacked the specialist skills required to deal with pervasive trauma. Complementing the metaphor of a cult, participants commonly framed their recovery journeys as one of deprogramming while navigating the complexities of the shame associated with the individual having “willingly consented” to conversion practices, and loss of faith; a significant component of their identifies. Consequently, participants often disengaged or described their experiences engaging with medical or psychological support as “entrenching the damage”.

*If I was going to go to a doctor, therapist, or a psychologist I would be looking for someone who understands cult dynamics, is trauma trained, knows about PTSD, and understands the self-doubt and absolute feeling of being alone that many of us share. (Participant #12)*

Next, accessing psychological support was often cost prohibitive. This was especially noted for those who were not currently able to engage in regular paid work and were reliant on government assistance. Participants repeatedly drew attention to government funded mental health support for survivors of sexual abuse through Accident Compensation Corporation (ACC) and shared frustration that spiritual abuse survivors are ineligible for these services (Accident Compensation Act 2001).

### 3.6.Pipeline to safety and recovery

Spiritual abuse was analogously aligned to family and sexual violence, and participants variously enlisted the metaphor of a pipeline to describe a multidimensional set of interventions required to ensure those entrapped within spiritual abuse have a “pipeline to safety”. Significantly, the pipeline needs to provide support to survivors while they are entrenched within abusive religious settings, immediately after they have left abusive environments, and throughout their healing journeys.

*We need to be able to get victims out of families and environments that are abusive. They need to know that they can leave, and they need to know how to leave. I am thinking something like a Women’s Refuge model. (Participant #3)*

#### 3.6.1. Establishing a dedicated agency to support spiritual abuse survivors

Under the rubric of abuse, participants stressed the need for government recognition of conversion practices as spiritual abuse and the concomitant need for support to assist survivors leave abusive settings and support their healing journeys. Within this context, participants suggested the need for a dedicated body to be established to support spiritual abuse survivors.

Participants differed according to whether the establishment of a dedicated body should be in the form of a government agency, or specialist non-government agencies funded by the government, to respond to the needs of survivors. The form of the agency, whether State or non-government, was, however, of less concern than the need to develop an intervention and support mechanism that is fully cognisant and responsive to the needs of survivors. It was noted that such interventions needed to be separate from existing child protection interventions which have resulted in additional harm to the child.

*We need something that is about the survivor. It cannot be built on the systems that we currently have, which often do more harm than good. (Participant #4)*

Further, participants stressed that the agency should have a singular focus on engaging and supporting survivors, in accordance with survivor needs and timeframes. It was especially noted that interactions need to avoid re-victimising the individual. As such, it cannot be assumed that all survivors want, or are ready, to leave the abusive environment. Rather, the priority is providing the individual with choice and the knowledge required to leave once the decision has been made.

*And not all kids want to leave. You need to create options for people so they can make informed choices. It is about recognising that they [survivors] don’t want people to take their control away. If they put their hand up and say something’s wrong, that doesn’t mean they’re handing it over to you to do what you think needs to happen. They need you to walk alongside them and find the pathways they want. (Participant #11)*

#### 3.6.2. Ensuring survivors’ basic needs are met

The provision of housing, financial support, and education and training opportunities was forwarded as essential to enabling survivors to leave abusive environments. Similarly, participants stressed the necessity for the provision of accessible, cost neutral, trauma-informed medical care and psychotherapeutic support and it was strongly suggested that ACC legislation be amended to include spiritual abuse.

While there is an urgent need for government agencies to appropriately support spiritual abuse survivors, participants also stressed that a number of faith settings have indicated a willingness to support survivors who have been abused within religious settings, generally through the provision of housing and a supportive community. Within this context, participants suggested the need for the State to collaborate with survivor-aligned faith settings as many participants reflected that support from those who understand their faith would have greatly assisted their recovery.

#### 3.6.3. Raising awareness and engaging survivors

Raising awareness about spiritual abuse was posited as integral to the development of a pipeline, as many participants had not realised that they had been experiencing conversion practices; only appreciating the normalised nature of the abuse well after they left the abusive environment. Within this context, participants recommended widescale promotion of the nature and impact of conversion practices along with information about where the individual might receive support.

*I didn’t realise it was conversion therapy. When you don’t know what you are experiencing is wrong, and when you don’t have the words for it, you don’t know you need help. (Participant #13)*

School-aged children were raised as a particular concern and participants stressed the need to first acknowledge schools as an important dual nexus for information promotion and intervention.

*There should be signs up at school saying, ‘If you’re having these experiences there are ways of getting help”. And we need to make it safer for children to talk. (Participant #14)*

An outstanding concern were children and youth who are either schooled in private religious school settings or in the home. Participants recommended the need for targeting planning on how awareness-raising, and help-seeking options, might occur in these settings.

#### 3.6.4. Establishing intervention pathways

Considerable discussion centred on the need to inform and support those who are best positioned to potentially identify spiritual abuse and support the individual should they choose to leave an abusive environment. Those supportive of an intervention pathway cited the need for training and awareness-raising on indications of spiritual abuse for teachers, members of faith communities, nurses, and general practitioners. A more complicated, and unresolved issue, however, is the need to establish intervention pathways that avoid retraumatising the individual.

*None of the professionals I met were empowered in any way to make any material difference to the circumstances of my life. None of my teachers. Not my doctor. None of the adults in my church. I don’t think they knew what the options were and so they didn’t do anything. (Participant #13)*

#### 3.6.5. Medical and psychotherapeutic support

Participants stressed that there is an urgent need for professionals, namely nurses, medical providers, and those who provide psychotherapeutic support, to be sufficiently trained in the dynamics of spiritual abuse, the effects of living in cult-like environments and to develop the requisite skills to appropriately support survivors. Especially stressed was the need for professionals to be trauma-informed and trained. Continuing education modalities were suggested for nurses and medical providers, exposing the learners to the dynamics and effects of spiritual abuse. Specialist training on spiritual abuse, recovery from cult-like environments, and the association with post-traumatic stress disorder for psychotherapeutic practitioners was strongly recommended. It was further recommended that professionals who have been sufficiently trained in spiritual abuse are appropriately promoted to ease identification. Some participants noted, those working in helping professions need to be aware of dynamics of power and privilege and the associated risk of further marginalising individuals who have experienced trauma. Within this context, there are benefits in exploring psychotherapeutic models with a social justice foundation [44, 45].

Specific commentary centred on faith-based counsellors, and concern was raised that there are no safeguards surrounding what faith-based counselling entails and the fact that it exists outside of a registration pathway. Consequently, participants suggested the need to require professional registration and ensure the adequacy of training. Above all, however, those identifying as faith-based counsellors should be required to refer a client that falls outside of the heteronormative dictates of the faith, to an objective third party.

#### 3.6.6. Establishing supportive communities

All participants stressed the importance of supportive communities and social networks in facilitating their recovery journeys and stressed the need for peer-led networks to be established, promoted, and supported as determined by survivors. Participants suggested that the establishment and support of peer-led networks could be facilitated by the newly established survivor agency and survivor-aligned faith settings.

*Access to other people who have had similar experiences has been incredibly beneficial; not feeling like you’re the only one that’s kind of gone through something. I think there is validation and healing and recovery in shared experiences. (Participant #14)*

#### 3.6.7. Preventing harm within faith-based organisations

A high degree of caution was shared that conversion practices legislation, as the only current existing intervention, would not have a significant impact on fundamentalist faith environments. Specifically, some of these settings were reported as circumnavigating legislation by obfuscating formal conversion practices. Similarly, participants were unanimously concerned that the legislation fails to address the existence and traumatic impact of informal conversion practices and conversion ideology.

While participants suggested it would be beneficial to explore possible legislative amendments, such as the inclusion of conversion ideology, there was consensus that shifts away from abusive practices are more likely to occur through dialogue with fundamentalist faith settings; the aim of which is to shift attitudes by increasing understanding of the impact of conversion practices on those who fail to meet heteronormative and traditional cisgender conceptualisations. Some participants felt that greater understanding of the risk of suicidality, self-harm, and mental distress and information about various support options might increase the possibility of pathways to facilitate wellbeing. Moreover, it would be especially advantageous for faith members to be encouraged to seek support from someone external to the faith community.

*Churches don’t want their children to die. If we pointed out the downstream impacts are incredibly dangerous for your children, then they’d have to think about how to best support and not harm. We have to be able to have a conversation where we talk about what safety means and how there are ways that people can have faith, have their beliefs, and can still stop the children being so damaged in the process. (Participant #3)*

## 4. Discussion

Within the context of post-conversion practice legislation, this study identified the potential for unique holistic intervention and support opportunities for survivors of religious conversion practices; opportunities that address the intersection of mental, physical, and spiritual health at an individual, whānau, community, and societal level. Moreover, rather than viewing conversion practices at a simplistic level of individual harm, conversion practices are framed as spiritual abuse that occurs in environments of harm. Appreciating conversion practices as abuse appropriately frames coercive control and entrapment as pervasive mechanisms that prevent the identification of conversion practices as abuse, impede disengagement from conversion practices, and create barriers to leaving an abusive religious context and engagement in independence and recovery. The continued description of conversion practices as ‘harm’ risks minimising their impact. Conceptualising conversion as spiritual abuse elevates conversion practices from a minimising discourse of harm and positions conversion as requiring urgent intervention and ongoing support, inclusive of the development of policy and operational responses.

Life history narratives highlight the spiritual, social, and structural entrapment experienced by those subjected to formal and informal conversion practices, regardless of the contexts in which participants experienced conversion practices. Intervention and support opportunities need to be responsive to each of these layers of entrapment. Analogously aligned to family and sexual violence, the metaphor of a pipeline was invoked to ensure those entrapped within spiritual abuse are provided a “pipeline to safety”; a metaphor that embodies extraction pathways and support for those entrenched within abusive religious settings, support immediately after leaving abusive environments, and support throughout the survivors’ healing journeys.

While limited to survivors in Aotearoa New Zealand, this study’s findings are relevant to other jurisdictions and conversion practices research, advocacy in general and practices; beliefs that were commonly framed as “fundamentalist”; a reference to a belief in biblical infallibility. Given the lack of consensus surrounding various conceptualisations of conversion practices, there is a need for dialogue within the research and advocacy communities about how we might be unified in incorporating information about the prevalence, experiences, and needs of those whose sexual orientation, gender identity, and gender expression position them in opposition to heteronormative dictates. Further, the inclusion of religious and non-religious conversion practices is vital. It is essential, however, to treat the experiences as distinct. We argue that failure to do so risks conflating experiences, which effectively risks ignoring lived experiences and opportunities for intervention and support that are unique to each cohort.

As jurisdictions consider legislation that prevents conversion therapy, this study’s findings issue a challenge to critically engage with the existence of conversion practices at an informal and ideological level while ensuring that legislation addresses survivor needs through the creation of interventions and the provision of ongoing support. Failure to do so risks continued harm. Significantly, a level of compassion was evident throughout participants’ narratives. Rather than vilifying perpetrators, participants were cognisant that there is an urgent need to end spiritual abuse while simultaneously supporting people’s right to exercise their faith. Such compassion is most keenly evident in the emphasis participants placed on dialogue; an emphasis that acknowledges that shifts away from abusive practices are more likely to occur through dialogue with fundamentalist faith settings. Such compassion appears to be founded on an understanding that legislation that places the onus on survivors to make complaints is problematic, as spiritual abuse occurs in settings that often include friends and family who collude with the various mechanics of abuse. In this context, requirements to report spiritual abuse can act as a significant barrier as reporting risks the potential criminalisation of loved ones.

## 5. Conclusions

While conversion practice-related prevalence and harms have been well established there is an urgent need to develop holistic interventions and supports for survivors. Drawing upon a metaphor of a pipeline to safety, responses to spiritual abuse are best informed by an understanding of entrapment. Holistic responses are needed to provide extraction pathways and support for those entrenched within abusive religious settings, support immediately after leaving abusive environments, and the provision of support throughout the survivors’ healing journeys. Engagement and education in these settings is required to address, and ultimately end, the use of conversion practices.
